# The HIV/AIDS responses pre and during the COVID-19 pandemic in sub-Saharan Africa: A basis for sustainable health system strengthening post–COVID-19

**DOI:** 10.1016/j.ijregi.2023.09.006

**Published:** 2023-09-26

**Authors:** Derek Mangoya, Enos Moyo, Grant Murewanhema, Perseverance Moyo, Itai Chitungo, Tafadzwa Dzinamarira

**Affiliations:** 1 The Centre for HIV and AIDS Prevention Studies, Windhoek, Namibia; 2 University of Kwa-Zulu Natal, College of Health Sciences, School of Nursing & Public Health, Durban, South Africa; 3 Faculty of Medicine and Health Sciences, University of Zimbabwe, Harare, Zimbabwe; 4 Medical Centre Oshakati, Oshakati, Namibia; 5 School of Health Systems and Public Health, University of Pretoria, Pretoria, South Africa

**Keywords:** HIV/AIDS response, Successes, Sub-Saharan Africa, COVID-19, Future pandemics, Health system strengthening

## Abstract

In May 2023, the World Health Organization announced that COVID-19 was no longer a global emergency. The impact of COVID-19 on the provision of HIV/AIDS services was not that severe due to the reprogramming of key resources. For health systems in sub-Saharan Africa to become resilient to future pandemics, lessons should be learned from the successful HIV/AIDS response in the region, and how the HIV/AIDS services were successfully adapted to the COVID-19 pandemic. In this article, we reviewed the 6 World Health Organization health system building blocks on how the best practices from the provision of HIV/AIDS services and the services’ response to the COVID-19 pandemic can be used as a basis for restoring and strengthening health systems to offer universal access to quality essential health services. The success of the leadership and governance for universal access to anti-retroviral therapy can be a blueprint for the realization of universal health coverage. Significant efficiencies that resulted in the reduction in anti-retroviral therapy costs can be leveraged to ensure cheaper essential drugs while differentiated service delivery models can be used to improve health service accessibility. New technologies that have proven to be successful in HIV/AIDS care can also be used in the care of other diseases, including disease outbreaks. The strong health information systems developed for HIV programs can be used as a foundation for developing health information systems for the whole health sector while the healthcare professionals trained for the provision of HIV/AIDS services can be trained to provide services for a variety of other conditions.

## Highlights

• Authors explored successes in HIV response pre and during COVID-19.
• HIV anti-retroviral therapy coverage, differentiated HIV service delivery models, and technological innovations are discussed.
• Tailored approaches for sustainable health systems strengthening are discussed.

## Introduction

In the first week of May 2023, the world breathed a sigh of relief with the announcement by World Health Organization (WHO) that COVID-19 was no longer a global emergency [1]. While this does not signify the end of the pandemic, it gives the world time to take stock of the factors that continue to make health systems, particularly in sub-Saharan Africa (SSA), vulnerable to outbreaks. The announcement coincided with the publication of a report revealing that the disruption to routine health services globally had significantly dropped to 25% of all essential services in the first quarter of 2023 signifying a near return to pre-COVID-19 health services delivery [2].

While at the time of this announcement Africa had recorded the least number of confirmed cases, 10 million of the world's 765 million, and 175 000 of the 7 million total global deaths, the same cannot be said about the disruption to routine essential services [3,4]. It is needless to point out that the total reported cases in SSA were possibly grossly underestimated owing to the limited testing capacity highlighting fundamental flaws in pandemic responses [5].

While the full impact of the disruption of individual services is not yet clear, several lessons learned during the pandemic will be needed to transform the healthcare sector into a more resilient system. Lessons from HIV/AIDS services during COVID-19 and the resilience of the HIV/AIDS epidemic response throughout the years would be invaluable. Although the COVID-19 pandemic disrupted health services including key HIV services, the organized and resilient response to the HIV epidemic allowed for the reprogramming of key resources including funding, human resources, laboratory services, and health information platforms for better delivery of the emergency pandemic response. The HIV/AIDS epidemic response also taught enduring lessons which will leave health systems stronger not only for future pandemic responses but also for universal health coverage. In this paper, we review the 6 WHO health system building blocks on how the best practices from the provision of HIV/AIDS services and the services’ response to the COVID-19 pandemic can be used as a basis for restoring and strengthening health systems to offer universal access to quality essential health services.

## Health systems strengthening post–COVID-19

The response to COVID-19 impacted the HIV/AIDS response in SSA which is already home to the largest population on treatment and has countries with the highest incidence of HIV. Global advice for social distancing and restriction in movement meant innovative ways to ensure uninterrupted care and treatment were necessary [3]. As patients shunned hospitals for fear of contracting COVID-19 and lockdowns restricted access to health centers and attention focused on COVID-19 screening, testing, and treatment, key innovations, some of which were already in the pipeline, had to be rolled out. Key to the adaptation of HIV/AIDS programs to the COVID-19-induced disruptions are the global, regional, and local structures supporting health systems’ delivery of HIV/AIDS services [6].

### Leadership and governance

The HIV/AIDS epidemic elicited a global response to a health emergency like no other until the recent COVID-19 outbreak. The response, starting with political leadership commitment, embraced all considerations from human rights, equality, gender sensitivity, and minority involvement, with a resultant unmatched global solidarity driving an unprecedented global resource mobilization to achieve universal access to life-saving anti-retroviral medication [7]. Out of this, the global coordinating mechanism, UNAIDS was born, and the resultant accountability demand resulted in the governance structure across all levels that shape the resilience of the response even today. HIV/AIDS coordinating structures have existed not only at parent ministry levels but across various global and regional agencies, multiple government ministries, and multiple stakeholders at every structure of governance within countries [7]. According to the UNAIDS, globally, by the end of 2021, 85% of people who were infected with HIV knew their status, 88% of the HIV-positive people were on treatment and of these 98% had virological suppression [8]. The new ambitious 95/95/95 target by 2025 should carry the world toward declassifying HIV/AIDS as a global public health threat by 2025. The success of the leadership and governance in universal access to anti-retroviral therapy (ART) can be a blueprint for the realization of universal health coverage (UHC) as part of global commitment to goal 3 of the Sustainable Development Goals (SDGs).

### Health financing

Vast financial resources have been used in the response to HIV/AIDS with foreign aid sustaining the health sector response in the early years. For 2025, global financial commitment to end HIV/AIDS as a global public health threat is projected to be a staggering US$ 25 billion with UNAIDS reporting that 60% of the US$ 21 billion used in 2021 was from domestic spending [8]. However, for 50% of the SSA countries, domestic expenditure accounts for 25% or less of the total expenditure on HIV/AIDS. Increasingly, the donor community is pressing ahead with plans for sustainable integration of HIV/AIDS into health systems coupled with funding cuts. The initial vertical programming of HIV/AIDS responses and Official Development Assistance (ODA) for specific programs has been blamed for creating donor dependence with central governments not mobilizing enough resources or disinvesting when ODA is available [9,10]. This means that ODA does not become additional to local funding for health but is used to substitute what would have been allocated from domestic sources.

Supporting global public goods (GPG) as an alternative to direct development assistance for health is considered less fungible [11]. Compared to other disease areas like non-communicable diseases and maternal and child health, significant efficiencies have been found that have reduced the costs of HIV care and treatment. Through GPG support, HIV/AIDS as a global sector has been able to force price reduction of drugs, equipment, and consumables through negotiated commercial agreements and pooled procurements [11]. The large global cohort of HIV/AIDS, patients with tuberculosis, and vaccine-eligible populations have also offered sufficient incentives for market shaping to reduce prices [11], and the same can be done across all healthcare sector needs.

### Service delivery

ART services built very efficient outreach models as a way of reaching out to individuals in need of care through testing, initiation of treatment, follow-up, and screening and treatment of opportunistic infections [12]. The sustained high number of people needing ART follow-up due to the success of HIV treatment services and revised guidelines recommending treatment for every newly diagnosed patient stretches the care cascade. The health systems in SSA have been asked to do a lot more for far less as global financial support wanes and efforts are being made to wean HIV care off donor dependence [10].

To maintain quality services differentiated HIV service delivery (DSD) models which emphasize patient education and empowerment which were being pioneered in several countries at the time when COVID-19 broke out proved a stroke of genius in limiting the vulnerable patients with HIV to the highly infectious COVID-19 [3]. The models triage patients into the category of those in need of intense facility-based management and those who are stable and do not need frequent facility visits.

DSD established new efficiencies in that the healthcare workers available can see fewer patients while maintaining the quality of care [3]. HIV chronic medicine delivery through DSD models showed no difference in outcomes compared to standard traditional care [13]. While all health systems integrated COVID-19 screening at the peak of the pandemic, the HIV continuum of service had also started integrating non-communicable diseases screening and treatment as routine services [14].

### Medical products, vaccines, and technologies

The digital industry gained pre-eminence during the COVID-19 pandemic with virtual platforms allowing for virtual engagement for business and education. Rapid infrastructure development and adaptation of available telecommunication systems have not spared the health delivery system. While mHealth is being tried in multiple settings in Africa, a systematic review of trials, most of which involve HIV care and treatment, showed some benefit of two-way text messaging (messaging patients with the option of them to respond) in improving treatment adherence but the results were inconclusive when it came to attending follow-up [15].

The efficiency gains from such interventions cannot be doubted and one cost-effectiveness study of two-way texting follow-up after Voluntary Male Medical Circumcision (VMMC) for HIV prevention in Zimbabwe was dominant in the incremental analysis when compared with the usual standard physical follow-up [15]. Telehealth was considered a long shot but a rapid shift to embrace digital communication for treatment follow-up in HIV programs has been documented and the impact is being evaluated [15]. The evidence from the innovation during the COVID-19 pandemic needs to inform strategies for the restoration of essential health services.

With the slow penetration of digital technologies in Africa, it remains to be seen how successful digital health for non-contact consultation has been as health systems restore services. Opportunities for the use of artificial intelligence including chatbots for screening patients should be opened to patients who can benefit from such. While community pickup points have existed for more than a decade now as early precursors to DSD, newer innovations include unmanned smart lockers which can harness existing mHealth principles to send unique access codes or apply artificial intelligence to deliver medication to individuals that the technology can positively identify at locations closer to them [16].

One key milestone for Africa in the fight against COVID-19 was the detection of a new variant of the disease by African researchers in Botswana [17]. The Omicron variant went on to become one of the most dominant strains and its discovery was premised on the use of HIV infrastructure that had been intentionally reprogrammed to expand the diagnostic and surveillance capability not only in Botswana but the majority of the SSA countries with high HIV burden. The laboratory polymerase chain reaction capacity for HIV and tuberculosis testing and follow-up was quickly adapted to include polymerase chain reaction COVID-19 testing. The rapid development of a vaccine for COVID-19 was built upon 40 years of HIV research and development including the infrastructure, synergies, and collaborations to support the pivoting to rapid COVID-19 vaccine development [18].

### Health information

Health information routinely collected is valuable for informing population health needs, tracking outcomes, quality improvement, and supporting research. While there has been significant improvement in Routine Health Information Systems (RHIS) across SSA, the quality is still very poor [19]. The information is usually of poor quality, the data aggregated and not available on time for use in forecasting and tracking individual patients [19].

The HIV programs have been supported to build individual disaggregated RHIS which made it easier to track outcomes for individual patients and supported variations in HIV service delivery during disruptions during the COVID-19 pandemic. The good practices in HIV RHIS have already been noted to have had a snowball effect across the generality of health systems. Across different health program areas, the majority of RHIS in SSA remain fragmented posing a challenge to the sustainable and integrated restoration of health services [19]. The core strengths in the RHIS in HIV program and those developed for COVID-19 need to be integrated and support the decision toward building electronic source records to ease communication and processing of health information within health systems and support digital health.

### Health workforce

The HIV/AIDS response recognized the key human resources needs for a highly skilled and motivated workforce. Medical doctors, at first specialists, were at the forefront of HIV/AIDS management as they manned the HIV opportunistic infection clinics and were solely responsible for the initiation and follow-up of patients. Health systems in SSA which have a limited training capacity and are greatly impacted by brain drain, have evolved, and embraced task shifting with HIV services perhaps offering the best lessons in technical efficiency [7]. To date, Primary Health Care (PHC) services in HIV high-burden countries have integrated HIV care into their primary healthcare basket of services and allow ease of conformity to the guidelines of test and treat, which is the initiation of same-day treatment for ART-eligible clients [10]. This is also allowing HIV care and treatment to graduate from vertical programs to fully integrated services. The availability of a lot of human resources for health (HRH) in the HIV response allowed to some extent minimal disruption of services due to the COVID-19 quarantine procedures and absence due to illness. It also allowed highly skilled workers to attend to needy patients and the redeployment of some health workers to the COVID-19 response [20].

## Conclusion

With the removal of COVID-19 as a global health emergency and the reported healthy recovery of essential health services to pre-COVID-19 levels, sustainable health systems can be built around the framework of the HIV/AIDS epidemic response. The strengthening of health systems across Africa post–COVID-19 will rely a lot on embracing PHC initiatives which offer affordable, effective, and efficient ways to tackle many of the challenges in SSA. PHC re-engineering is recognized by many countries as a vital strategy in the UHC journey for SSA. Stronger, resilient health systems built on a backbone of PHC will limit the pressure of tertiary facilities allowing the channeling of key resources to the management of those in need of tertiary care. 40 years into the HIV/AIDS epidemic, with epidemic control in sight, the lessons from the HIV/AIDS epidemic response will be invaluable.

## CRediT authorship contribution statement

**Derek Mangoya:** Conceptualization, Writing – original draft. **Enos Moyo:** Conceptualization, Writing – original draft. **Grant Murewanhema:** Writing – review & editing. **Perseverance Moyo:** Writing – review & editing. **Itai Chitungo:** Writing – review & editing. **Tafadzwa Dzinamarira:** Writing – review & editing.

## Declarations of Competing Interest

The authors have no competing interests to declare.
